# Nuclear Medicine and Cancer Theragnostics: Basic Concepts

**DOI:** 10.3390/diagnostics13193064

**Published:** 2023-09-26

**Authors:** Vasiliki Zoi, Maria Giannakopoulou, George A. Alexiou, Penelope Bouziotis, Savvas Thalasselis, Andreas G. Tzakos, Andreas Fotopoulos, Athanassios N. Papadopoulos, Athanassios P. Kyritsis, Chrissa Sioka

**Affiliations:** 1 Neurosurgical Institute, University of Ioannina, 45110 Ioannina, Greece; 2 Department of Neurosurgery, University of Ioannina, 45110 Ioannina, Greece; 3 Institute of Nuclear and Radiological Sciences and Technology, Energy and Safety, National Center for Scientific Research “Demokritos”, 15341 Athens, Greece; bouzioti@rrp.demokritos.gr; 4 SYN Innovation Laboratories SA, 14451 Athens, Greece; 5 Department of Chemistry, Section of Organic Chemistry and Biochemistry, University of Ioannina, 45110 Ioannina, Greece; 6 Department of Medical Physics, University Hospital of Ioannina, 45500 Ioannina, Greece; thanospapadopoulos400@gmail.com; 7 Department of Nuclear Medicine, University of Ioannina, 45110 Ioannina, Greece

**Keywords:** theragnostics, nuclear medicine, thyroid cancer, neuroendocrine tumors, prostate cancer, colorectal cancer, cholangiocarcinoma, glioma

## Abstract

Cancer theragnostics is a novel approach that combines diagnostic imaging and radionuclide therapy. It is based on the use of a pair of radiopharmaceuticals, one optimized for positron emission tomography imaging through linkage to a proper radionuclide, and the other bearing an alpha- or beta-emitter isotope that can induce significant damage to cancer cells. In recent years, the use of theragnostics in nuclear medicine clinical practice has increased considerably, and thus investigation has focused on the identification of novel radionuclides that can bind to molecular targets that are typically dysregulated in different cancers. The major advantages of the theragnostic approach include the elimination of multi-step procedures, reduced adverse effects to normal tissues, early diagnosis, better predictive responses, and personalized patient care. This review aims to discuss emerging theragnostic molecules that have been investigated in a series of human malignancies, including gliomas, thyroid cancer, neuroendocrine tumors, cholangiocarcinoma, and prostate cancer, as well as potent and recently introduced molecular targets, like cell-surface receptors, kinases, and cell adhesion proteins. Furthermore, special reference has been made to copper radionuclides as theragnostic agents and their radiopharmaceutical applications since they present promising alternatives to the well-studied gallium-68 and lutetium-177.

## 1. Introduction

**Figure 1:**
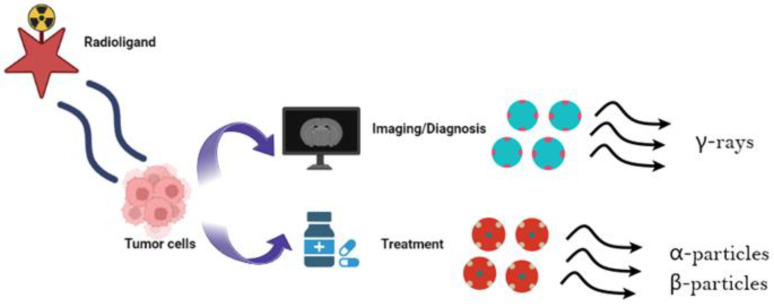
The use of radiopharmaceuticals as theragnostic tools. Depending on their decay properties, radioisotopes can possess either diagnostic or therapeutic capabilities. Specifically, γ-emitters may be utilized for diagnostic SPECT and PET imaging [5]. In contrast, α- and β-radionuclides that emit low-range, but highly ionizing radiation are specific for therapeutic purposes [6,7]. One commonly used theragnostic agent is iodine-131 (^131^I), for diagnosis and treatment of patients with differentiated thyroid cancer [8,9,10]. Several other commonly used theragnostic pairs include ^68^Ga/^177^Lu, ^43^Sc/^44^Sc/^47^Sc, ^83^Sr/^89^Sr, ^86^Y/^90^Y, ^110^In/^111^In, ^90^Y/^177^Lu, and ^152^Tb/^161^Tb [11]. Image created using Biorender (https://www.biorender.com/ accessed on 22 August 2023).

Cancer is a primary cause of death globally. Given its recurring and lethal nature, its cure remains unsuccessful for most patients. In recent years, there has been a great expansion of theragnostics, which consists of a comprehensive therapeutic process that encompasses identification of the cancer using a specific radioactive molecule that binds to the tumor, followed by the administration of a similar radioactive molecule designed to kill the malignant cells (Figure 1). In addition, another diagnostic post-therapy scan is usually performed that confirms the therapeutic response of the selected sites [1]. In certain cases, nuclear theragnostic agents with comparable molecular features are utilized, whereas in others, theragnostic compounds that are not biologically similar but have equal biodistribution are used [2]. The rapidly evolving field of theragnostics includes some already approved treatments such as ^177^Lu-PSMA (prostate-specific membrane antigen) for prostate cancer, ^223^Ra for osseous metastases, ^177^Lu-DOTATATE for neuroendocrine tumors, ^131^I for thyroid cancer, and several others that are under development [3]. The concept of theragnostics was initiated during the early days of nuclear medicine.

One such example represents the administration of iodine-131 followed by SPECT to diagnose thyroid cancer and the subsequent administration of a higher dose of the same radioactive molecule to attack and extinguish the cancer. However, during the last two decades, there has been tremendous progress in the actual construction and development of theragnostic molecules for personalized cancer diagnosis and therapy [4]. Examples of such recent molecules include [^68^Ga/^177^Lu]-labeled somatostatin peptides for theragnostics of neuroendocrine tumors. A similar molecule, [^68^Ga/^177^Lu] PSMA, may be used for metastatic prostate cancer. The exploitation and the clinical utilization of the range of diagnostic and therapeutic radioisotopes presupposes a deep knowledge of the physics of radiation. For an introductory section, the physical characteristics and the features of the decay schemes of the theragnostic radionuclides that are utilized today are presented in Table 1. Most of the presented radioisotopes are well known, since the large majority of them have been exploited in numerous applications—though not necessarily in clinical areas.

Ιt is essential to mention that the precise knowledge of the interaction of radiation with matter for each isotope lies behind a successful therapeutic approach. The use of different radiation modes, Auger electrons, α-emitters, and β-emitters can cause the desired death of cancer cells. We briefly recall that Auger electron emission causes double-strand DNA damage by direct internalization or through an indirect effect caused by the generation of free radicals due to water hydrolysis. The α-emitters with high LET cause high-density ionization effects, resulting in double-stranded DNA breaks. In contrast, the β-emitters mainly cause repairable single-strand DNA damage. The higher path length of β-emitters is approximately equal to 1000 cell diameters, resulting in crossfire radiation, whereas the Auger electron and α-emitters result in less cross-fire radiation due to their shorter path length.

Though these applications are very promising, there are significant practical challenges that must be solved for creative theragnostics to be implemented. For instance, the biodistribution of the theragnostic drugs should demonstrate adequate accumulation in the tumor, but very low concentration in the normal tissues; the diagnostic and therapeutic radionuclide half-lives must be appropriate for imaging and targeted cell killing, respectively; finally, the therapeutic radionuclides should be available to the patient within the time frame suggested by half-life and stability. In any event, recent evidence suggests that theragnostics is becoming an important contribution to cancer therapy [4]. In the present review, we summarize recent theragnostic molecules that have been investigated in various types of malignancies.

## 2. Theragnostics

### 2.1. Thyroid Cancer

**Figure 2:**
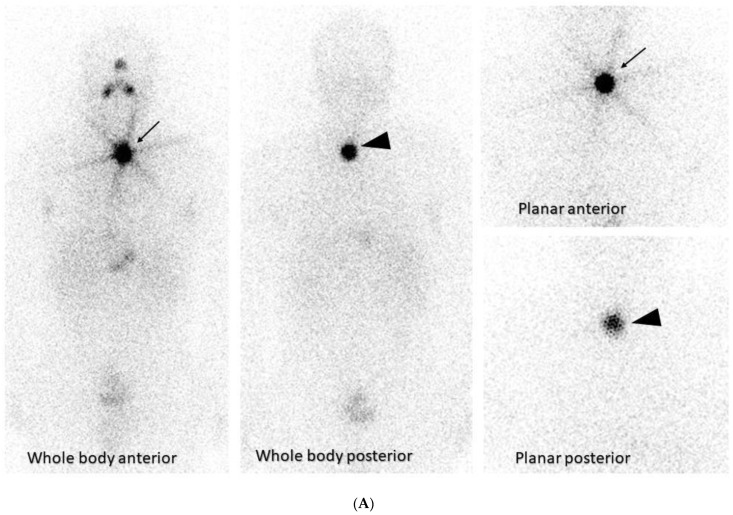

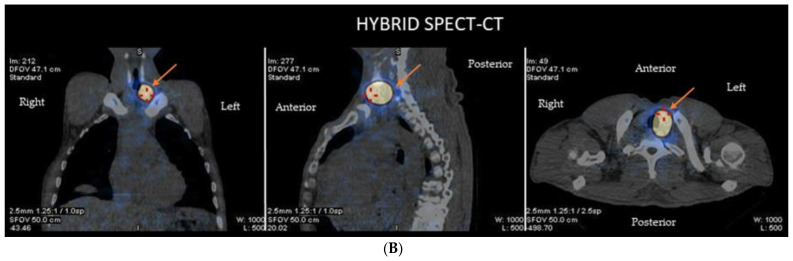
(**A**) A whole-body scan and planar images of a 42-year-old male with thyroid cancer who had initial radioactive iodine (RAI) therapy is depicted. The underlying principle of ^131^I thyroid therapy consists of the ablation of postoperative thyroid tissue residuals following thyroidectomy and treating metastatic disease after incomplete or total thyroidectomy. Dosing over 30 mCi results in obliteration of the thyroid by the beta particles causing ionization. This patient received 125 mCi ^131^I ablation therapy. Due to Hurtle cell carcinoma (oncocytic cell follicular thyroid carcinoma) in left lobe and 8 days post-therapy, whole-body imaging was performed to look for any nodal/distant metastases. A region of intense uptake was observed in the lower cervical region, on the left of the midline, giving an image similar to a star known as a “star sign” that is seen in the anterior images (this is due to the high uptake of RAI of the remnant thyroid tissue after surgery—arrow). The high uptake of ^131^I in the remnant thyroid tissue is also seen in the posterior images (arrowheads). No abnormal accumulation was noted elsewhere. (**B**) This figure demonstrates the hybrid single-photon emission tomography (SPECT) and low-dose computed tomography (CT) images for anatomical verification of the finding and disclosure of any nodal uptake near the star sign. The combination of CT with the iodine scan accurately localizes the thyroid tissue uptake (arrow) from the metastatic lesions, providing a three-dimensional view.

The usage of theragnostics in thyroid cancer comprises a combination of a diagnostic scan to localize the primary thyroid tumor and any metastases prior to surgical removal of the thyroid tumor and subsequent targeted ^131^I therapy to treat any remaining macroscopic or microscopic disease [12]. In fact, therapy with iodine-131 (^131^I) following thyroidectomy is one of the oldest theragnostic applications, introduced in 1946. It was based on sodium iodide symporter that thyroid cells express, which traps ^131^I and metabolizes it, aiding in the treatment of residual and metastatic disease [1] (Figure 2A).

The explosive development in the in nuclear imaging technology during the last several years, consisting of SPECT/CT and PET/CT systems, has allowed the achievement of precision diagnostic imaging, which localizes primary and metastatic tumors for remnant ablation or adjuvant ^131^I treatment of the tumor, avoiding the non-targeted healthy tissues [13,14] (Figure 2B).

^124^I PET/CT consists of an important imaging tool for the staging of differentiated thyroid cancer, but ^18^F-fluorodeoxyglucose (FDG) PET/CT is more precise for high-risk differentiated thyroid cancer or noniodine recurrences. However, poorly differentiated thyroid carcinoma is better evaluated by ^18^F-FDG PET/CT, or the ^68^Ga/^177^Lu-prostate-specific membrane antigen. As a new-generation imaging technique, novel molecular radiotracers based on PSMA ligand uptake evaluated with PET/CT have evolved. PSMA is a type II transmembrane glycoprotein that is extensively expressed in prostate cancer (PCa) and is the next imaging modality for PCa staging, re-staging, and response assessment [15]. When paired with ^177^Lu, it demonstrated greater diagnostic accuracy than traditional imaging in high-risk PCa patients, as well as treatment benefits (in terms of safety and efficacy). Despite its name, PSMA is not limited to the prostate; it has been reported to be expressed in the neovasculature of a variety of solid tumors, and enhanced uptake of ^68^Ga-PSMA PET/CT has been demonstrated in a variety of non-prostatic malignancies, including thyroid cancer [16,17]. Histological investigations, in particular, have confirmed the expression of PSMA in the microvasculature of thyroid tumors, where PSMA expression was associated with malignant disease, poor prognostic markers, and a lower progression-free survival (PFS) [18]. This evidence implies that PSMA PET/CT could be used as a theragnostic and prognostic imaging biomarker. Medullary thyroid carcinoma may be assessed by various PET tracers, such as [^18^F]-DOPA, [^18^F]-FDG, DOTA-octreotate (DOTATATE), and ^68^Ga-1,4,7,10-tetraazacyclododecane-1,4,7,10-tetraacetic acid [19,20]. For the diagnosis of distant metastases of medullary thyroid cancer, the use of [^68^Ga] Ga-DOTA.SA.FAPi had significantly higher sensitivity compared to [^68^Ga] Ga-DOTANOC PET/CT [21]. ^18^F-DOPA is a PET drug that targets the L-type amino acid transporter, which is expressed in both MTC and pheochromocytomas [22]. Though it is thought to be the most accurate method for detecting recurrent/metastatic MTC [23], particularly liver and cervical lymph node metastases, it is only available at specialist academic institutions. With an overall sensitivity of 47–83% [24], ^18^F-DOPA PET is most likely to detect uptake in MTC metastases in individuals with high calcitonin (>150 ng/mL). The sensitivity of ^18^F-FDG PET for MTC metastases is moderate (59–69%) [25], with the highest yield among individuals with short tumor marker doubling periods (one year). A study of ^18^F FDG PET indicated that in MTC with high calcitonin, the sensitivity was 92% and the specificity was 86%, with a significant impact on management decisions [26].

Finally, ^68^Ga-DOTATATE, a somatostatin analogue with strong affinity for SSTR2 that is widely available for neuroendocrine imaging, may be employed to screen for metastatic MTC. In comparison to other neuroendocrine neoplasms, this tracer has less dependable absorption, with relatively modest sensitivity for MTC (64%) [27], notably bone metastases. A recent study on ^68^Ga-DOTATATE PET indicated that it performed better than traditional imaging in 37% of 14/38 patients, changing patient care by finding neck nodes and bone metastases [28]. Despite this, detecting somatostatin receptor type 2 expression may qualify a patient with refractory disease for ^177^Lu-PRRT: a recent small series indicated that 62% of patients with verified DOTATATE uptake (27/43) demonstrated imaging evidence of disease response following PRRT [29]. Targeted novel PET drugs using Ga-68/Lu-177 theragnostic pairings, such as those targeting cholecystokinin-2 (CCK2R) receptors (DOTA-PPF11) and minigastrin (DOTA-MGS5), are also being studied. In any event, there are several radiotracers that aid in the effective diagnosis and therapy of the various types of thyroid cancers.

### 2.2. Neuroendocrine Tumors

Neuroendocrine tumors are typically characterized by increased expression of somatostatin receptors (SSTRs). Several clinical studies have demonstrated that targeting of the somatostatin receptor (SSTR) produced sufficient imaging for diagnosis and staging of neuroendocrine tumors. Furthermore, therapeutic intervention of these tumors includes treatment with either cold somatostatin analogs or radionuclide therapy against the somatostatin receptor. PET with somatostatin analogs labeled with ^68^Ga demonstrated high sensitivity for the diagnosis and staging of neuroendocrine tumors (NETs). In such patients, FDG PET/CT complemented molecular imaging with ^68^Ga-SSTR PET/CT [30]. A large study of 495 patients with metastatic neuroendocrine neoplasms used FDG PET/CT prior to treatment with peptide receptor radiotracer and found that the presence of positive lesions on FDG PET/CT was an independent predictor of outcome. The best long-term survival was associated with high expression of SSTR and negative FDG PET/CT [31].

Gastrointestinal NETs appearing in the intestines, appendix, or pancreas exhibit a variety of malignant transformations, with clinical activities depending on the mitotic activity calculated by the Ki-67 proliferative index and cell differentiation. The presence of SSTR expression in NET allows for SSTR imaging with ^68^Ga-DOTATATE PET/CT and radiotracer therapy against the somatostatin receptor. In addition, the ^68^Ga-DOTATATE PET/CT may be occasionally used for the staging and selection of patients for peptide receptor radionuclide therapy [32]. ^177^Lu-DOTATOC has also been studied as a promising therapeutic option for patients with metastatic bronchial NETs expressing high levels of SSTRs in a large study of 1200 patients [33]. In another recent study, in which patients with advanced midgut NET received ^177^Lu-DOTATATE and Sandostatin-LAR, longer progression-free survival and an overall survival benefit was observed for the combination compared to high-dose Sandostatin treatment alone [34]. The safety and efficacy of ^177^Lu-DOTATATE, as pointed out in different studies, has resulted in its approval by the FDA for the treatment of advanced SSTR-positive GEP neuroendocrine tumors [35].

In recent years, research has also focused on the use of alpha particle therapy with the radionuclides ^225^Ac, ^213^Bi, and ^212^Pb. The latter has been recently investigated in patients with metastatic NETsin a phase I clinical trial after being labeled with DOTAMTATE; however, the results of the study are not yet published [36]. The efficacy and safety profile of ^225^Ac-DOTATATE has been investigated in patients with metastatic NETs in terms of objective tumor response. The results showed that 37.5% of patients experienced stabilization of the disease, whereas the rest showed partial response [37].

### 2.3. Prostate Cancer

PSMA (prostate-specific membrane antigen), also known as glutamate carboxypeptidase II, is a zinc metalloenzyme encoded by FOLH1. It is localized in the cellular membrane. PSMA is expressed up to 1000 times more in prostate cancer cells compared to normal prostate cells, and the rate is even higher in advanced prostate tumors. Although PSMA does not represent a selective marker of prostate cells or malignancies, its pattern of expression in the prostate demarcates it as a theragnostic target for both imaging and therapeutic purposes [38]. Tumor characterization using PSMA ligand uptake for positron emission tomography (PET) imaging is important for subsequent PSMA radioligand therapy. Recently, the FDA approved two new drugs for PSMA PET imaging [39].

The radiopharmaceutical agent ^68^Ga-PSMA-HBED-CC (N, N′-bis-[2-hydroxy-5-(carboxyethyl)benzyl] ethylenediamine-N, N′-diacetic acid) represents a universal agent for PSMA-PET imaging. In this sector, a radiotracer targeting prostate-specific membrane antigen (PSMA) has showed interesting results. PSMA is a protein expressed in ondysplastic prostate cells at levels 100–1000 times greater than in normal cells, with levels increasing even more with higher stages and grades [40]. Recent meta-analyses, such as the one by Han S. et al., reveal that ^68^Ga-PSMA positron emission tomography (PET) offers outstanding diagnostic performance for primary and secondary staging due to its capacity to detect lesions even at very low serum PSA levels [41]. The following was the study question for this meta-analysis: “What proportion of patients experience change in their management when ^68^Ga-PSMA PET is used versus conventional imaging modalities (CT, MRI)?” Utilizing ^68^Ga-PSMA-PET for the initial staging of prostate cancer may result in the modification of initial treatment plans in a significant number of patients. The pooled proportion of patients experiencing a change in management was 54%. Similarly, ^68^Ga-PSMA-PET can detect patients with early recurrence, with higher sensitivity and specificity compared to conventional imaging modalities [42]. In addition, when new metastatic lesions were identified, ^68^Ga-PSMA-PET could suggest alternative treatment options for these metastases, such as stereotactic radiation therapy or surgical resection, resulting in better outcomes. Thus, this treatment approach may result in a reduction in anti-androgen therapy in some patients, avoiding its side effects [38]. For radioimmunotherapy, the diagnostic isotope is replaced by a therapeutic beta-emitting radioisotope coupled to the PSMA ligand. It is administered to the site of metastasis, where it binds to PSMA on prostate cancer cells and kills them. In a meta-analysis of nine clinical trials utilizing the ^177^Lu-PSMA-617 as the PSMA-radioisotope conjugate, an over 50% reduction in PSA levels in 37% of prostate cancer patients was shown [43]. Similar results of a 50% reduction in PSA levels in 44.2% of patients with castration-resistant prostate cancer were reported in another study of ^177^Lu-PSMA-617 therapy, as well as increased overall survival in responder patients in comparison to non-responders. In addition, there was noted improved radiologic progression-free survival and overall survival, as well as a better objective response rate and disease control rate [44]. Apart from beta-emitting isotopes, alpha-emitting radioisotopes such as ^213^Bi and ^225^Ac conjugated with PSMA-617 showed a favorable response in non-responders to ^177^Lu-PSMA-617 treatment [45].

FDG PET/CT has only limited use for advanced prostate carcinoma. Even though the PET radiotracer choline labeled with either ^18^F or ^11^C may detect early recurrence of prostate carcinoma, it has been replaced by other, more specific radiotracers [46]. Other radiotracers considered include bombesin analogs that showed a positive diagnostic rate of 71.8% in patients that exhibited a negative conventional imaging [47]. Bombesin analogues labeled with ^68^Ga can target the gastrin-releasing peptide receptors and can be used for PET imaging, with advantages in its availability, half-life, and relative low expenditure [48].

### 2.4. Colorectal Cancer

Although early non-metastatic colorectal cancer is curable, metastatic colorectal cancer usually represents an incurable tumor. Thus, early diagnosis prior to metastases remains crucial for a cure. Even though limited clinical data exist for these tumors after the appearance of metastatic lesions, characterization of genomic changes in metastatic lesions from the original tumor may modify a considered therapeutic intervention to a more personalized therapy. In such cases, a biopsy of the metastatic disease is recommended to examine any genomic alterations of the primary to metastatic tumor.

PET employing ^18^F-2-deoxy-2-fluoro-d-glucose (FDG) has emerged as a potential diagnostic method for recurrent colorectal cancer. The inclusion of FDG-PET affects disease management in up to 30% of patients with possibly resectable liver metastases, primarily by finding previously undisclosed extrahepatic illness, according to published data. Furthermore, because it is extremely sensitive in detecting residual or relapse malignancy in scarred liver tissue following both resection and local ablative treatments, FDG-PET is beneficial in the follow-up of patients who have undergone liver surgical procedures. Early FDG-PET appears to predict responsiveness to therapy during systemic therapy follow-up. For example, there are recent data showing that FDG-PET/MRI may change management in 19% of oligometastatic colorectal cancer cases [49]. Moreover, FDG PET/MRI enables local tumor evaluation and provides better N staging, particularly when evaluating low rectal tumors [50]. FDG-PET and computed tomography are complementary modalities for staging and restaging advanced colorectal cancer patients. The combination of these two approaches has a substantial impact on patient management [51].

### 2.5. Cholangiocarcinoma

Cholangiocarcinoma or bile duct carcinoma is an infrequent malignancy of the bile ducts. Cholangiocarcinoma may be intrahepatic (rare) or extrahepatic (more common). Extrahepatic cholangiocarcinoma is divided into perihilar cholangiocarcinoma, localized in the area where the right and left hepatic ducts join to form the common hepatic duct, and distal cholangiocarcinoma, located in the area where the common hepatic duct joins with the gallbladder cystic duct to form the common bile duct. Several biomarkers in the bile have been investigated as potential diagnostic and treatment targets because tumor fluid in that area is secreted directly into the bile and should possess markers that potentially can be identified. However, the mere presence of a biomarker in the vicinity of a tumor does not essentially qualify it as a molecule for theragnostics. The appropriate theragnostic biomarker should be in the tumor cell surface, to be able to bind easily with a systemically administered drug [52]. One such secretome biomarker present in the bile secreted by the cholangiocarcinoma is the neutrophil gelatinase-associated lipocalin (NGAL), a 25-kDa glycoprotein that forms a covalently linked complex with the 92-kDa type-IV gelatinase matrix metalloproteinase-9 (MMP9). NGAL possesses anti-microbial properties and is involved in the regulation of various types of cancer, such as stomach, colon, and pancreas cancer. The regulatory function of NGAL on cancer growth is mediated through its regulatory and stabilizing role on extracellular MMP9 and its anti-apoptotic effects on malignant cells. Thus, NGAL represents a biomarker that is overexpressed in the bile of patients with cholangiocarcinoma and in general in patients with any pancreato-biliary malignancy [53]. The production and secretion of NGAL in significantly larger amounts in cell lines of cholangiocarcinoma compared to normal cholangiocytes makes NGAL a potential candidate biomarker for use in theragnostic systems [54].

Recently, another interesting biomarker in cholangiocarcinoma is PSMA has been found for the first time in the tumor-associated neovasculature of most cholangiocarcinoma cases (79.3%) in a large sample set. PSMA expression was limited to the cholangiocarcinoma neovasculature, whereas normal liver and peritumoral tissues were mostly PSMA-negative. Although this is a premature finding, PSMA may have diagnostic power in cholangiocarcinoma and may be used as a treatment target [55].

### 2.6. Gliomas

Glioma is a type of tumor arising from glia. Glioblastoma (GBM) is the most malignant glioma, with a median survival time of affected patients of approximately 16 months. Diagnosis is based on imaging tools, mainly MRI, PET, and CT scan [56]. The current therapy for recently diagnosed malignant gliomas consists of maximal surgical resection if the tumor is accessible, followed by radiation therapy and chemotherapy [57]. Although temozolomide is the most effective chemotherapeutic drug, other chemotherapies may also be used upon failure of temozolomide. However, there are significant difficulties during chemotherapy treatment of gliomas, consisting of the presence of the blood–brain barrier, the diffuse structure of the neural tissue, and the presence of malignant cells within the glioma having multiple and different genetic abnormalities [58]. The blood–brain barrier hinders the entrance of most therapeutic drugs into the tumor, allowing only small, lipid-soluble substances to pass freely. In addition, the diffuse infiltrating structure of the tumor within the intermixed presence of normal glial and neuronal cells impedes chemotherapeutics to reach the vicinity of most tumors. Furthermore, most systemically administered antineoplastic drugs have modest pharmacokinetics and can also accumulate in healthy organs and thus increase the severity of side effects [59]. Finally, the presence of multiple and different genetic abnormalities within the same tumor make it difficult to treat, since some parts of the tumor may respond to a certain drug, whereas others are unresponsive, resulting in the development of resistance to chemotherapy [60]. Theragnostic agents based on radionuclides can play an important role in the management and treatment of gliomas, including glioblastoma, which remains the most aggressive type of primary tumor in the central nervous system. Several molecular targets have been explored for theragnostics in glioblastoma, including cell-surface receptors, kinases, and cell adhesion proteins, and other promising targets are implicated in different pathways affecting tumor growth, survival, and progression. Some of the most prominent targets for glioblastoma are summarized in Table 2.

In glioblastoma, radiolabeled small molecules are the most popular choices as potential theragnostic agents, since they present a more favorable pharmacokinetic profile and can cross the BBB. A molecular target that has been widely investigated for the development of this type of theragnostics is the enzyme PARP1, which is implicated in DNA repair. This enzyme is overexpressed in glioblastoma as opposed to non-tumoral cells. Since PARP becomes activated in response to DNA damage, inhibiting its actions pharmacologically or genetically may be a promising therapeutic option for cancers, including glioblastoma. Apart from the therapeutic potential, PARP inhibitors have also been explored as PET imaging agents. ^18^F-olaparib is a PARP-1 inhibitor designed in this accord, whereas another specific target, ^18^F-PARPi, primarily binds to peripheral tumors [70,71].

PSMA radioligands, primarily used in the management of prostate cancer, as mentioned before, have also gained attention as potential diagnostic tools for gliomas. Different studies have explored the efficacy of PSMA as a radioligand when combined with ^68^Ga. Nomura et al. were the first to report that grade IV gliomas showed increased PSMA staining [72]. The utility of ^68^Ga-68 PSMA molecules as a diagnostic tool has been recently examined by Kumar et al. in recurrent high-grade glioma patients. The results showed that this compound is a promising imaging tool for evaluating recurrence in glioblastoma [73]. In another study, the use of ^68^Ga-PSMA-11 brain PET/CT for the evaluation of recurrent glioma also showed increased PSMA uptake, and the absence of normal brain uptake resulted in much better visualization of glioma lesions [74]. Interestingly, when compared to conventional ^18^F-FDG PET, PSMA-targeting radiopharmaceuticals have the advantage of lower uptake in the normal brain [75]. However, despite the efficacy of PSMA-based molecules as diagnostic tools, there are still no clinical studies to evaluate their potential therapeutic role in glioma patients.

Chemokine receptor-4 (CXCR4) is highly expressed in different cancers, including gliomas, and is related to the neo-angiogenesis, migration, and survival of malignant cells. A preferential expression of this molecule by high-grade glioma cells and a relationship with poor patient survival have been previously reported [76]. In this regard, Lapa et al. made a radiolabeled pentapeptide showing high affinity to CXCR4, named ^68^Ga-Pentixafor, which was then tested as a PET imaging tool. Interestingly, the tracer showed high specificity towards high-grade glioma cells and presented a higher tumor uptake compared to popular tracers like ^18^F-FET [77]. CXCR4-directed anti-glioma therapy using cytotoxic radionuclides like ^177^Lu may thus be a promising therapy for glioblastoma.

Two promising radionuclides that can be conjugated with chelators and act as antibody-based theragnostics are ^89^Zr and ^177^Lu. Recently, Foster et al. developed a bifunctionalchelator, named Lumi804, to be used in combination with either of the metals mentioned above as a theragnostic agent in glioblastoma. This radiolabeled molecule targets the tumor microenvironment and may significantly improve immunotherapy [78]. Another promising radionuclide that has been explored for its PET imaging properties in glioblastoma is ^99m^Tc. When linked to tetrofosmin, ^99m^Tc-TF showed an increased uptake by glioblastoma cells. Given the fact that this radiotracer appears to be less affected by the existence of the elimination and uptake mechanism of chemotherapeutic substances, which certain cancer cells possess through p-glycoprotein, it may be a promising agent for the development of novel theragnostic agents through chelation with an appropriate cytotoxic agent [79,80].

### 2.7. Neuroblastoma and Other Pediatric Tumors

Neuroblastoma is a common extracranial malignancy in young children originating from neural crest progenitor cells [81]. A theragnostic approach for these pediatric patients includes iodine-labeled metaiodobenzylguanidine (MIBG). This norepinephrine analog has the ability to accumulate into the neuroendocrine cells by primarily utilizing the cells’ normal norepinephrine transporters. Another minor mechanism of MIBG cell uptake includes passive diffusion [82]. MIBG is labeled with ^123^I or ^131^I. In a past study, 13 children with advanced neuroblastoma were treated with ^131^I-MIBG combined with chemotherapy. The results showed that the addition of the theragnostic agent did not induce any additional toxicity compared to chemotherapy alone. Moreover, most patients responded very well to the combined treatment, and only one patient exhibited a mixed response [83]. In another study, in which pediatric patients with relapsed neuroblastoma were treated with high doses of ^131^I-MIBG, no significant toxicity was observed, whereas the overall survival rate 1-year post-treatment was measured at 58% [84]. Apart from neuroblastoma, MIBG has been used in other types of pediatric malignancies, including paraganglioma and pheochromocytoma. These rare tumors are primarily treated by surgery; however, in the case of diffuse metastases or non-accessible anatomical regions, the use of ^131^I-MIBG has shown promising results [85]. Apart from iodine-labeled MIBG, a PET tracer based on fluorine ([^18^F]-meta-fluorobenzylguanidine) has recently been designed. When 40 pediatric patients with a mean age of 6 years old and a history of neuroblastoma underwent [^18^F]MFBG PET/CT and [^123^I]MIBG SPECT/CT studies, a more favorable lesion detection rate was observed with [^18^F]MFBG PET/CT, implying that this radiolabeled agent may be a promising alternative to the well-studied ^123^I-MIBG theragnostic [86].

## 3. Copper Radionuclides as Theragnostic Agents

Copper radionuclides have been studied as promising theragnostic agents, and the most effective copper isotope appears to be ^64^Cu. Several efforts have been made to evaluate the potential of this radioisotope as a diagnostic PET/CT tracer in clinical oncology. The nuclear properties of ^64^Cu make it a great example of a theragnostic radionuclide and an interesting alternative to the well-studied gallium-68 (^68^Ga) and lutetium-177 (^177^Lu). In PET, the radionuclide decays through different routes. The resulting positrons are destroyed upon contact with electrons in the body, producing photons that can be detected and analyzed by computers to locate the exact source of the annihilation event [87]. Specifically, ^64^Cu is implicated in a complex decay scheme, involving three different processes. It can emit low-energy positrons, β^−^ particles, and Auger electrons. Copper-64 decays with a half-life of 12.7 h throughout positron emission (17.5% β^+^), beta emission (38.5% β^−^), and electron capture (44.0% EC), as shown in Table 1. Electron capture, in addition to characteristic X rays, is accompanied by the emission of high linear-energy transfer Auger electrons, which add to its cytotoxic potency if the radionuclide is located inside cells, particularly within or close to cell nuclei, given the short range of these low-energy electrons [88].

**Figure 3:**
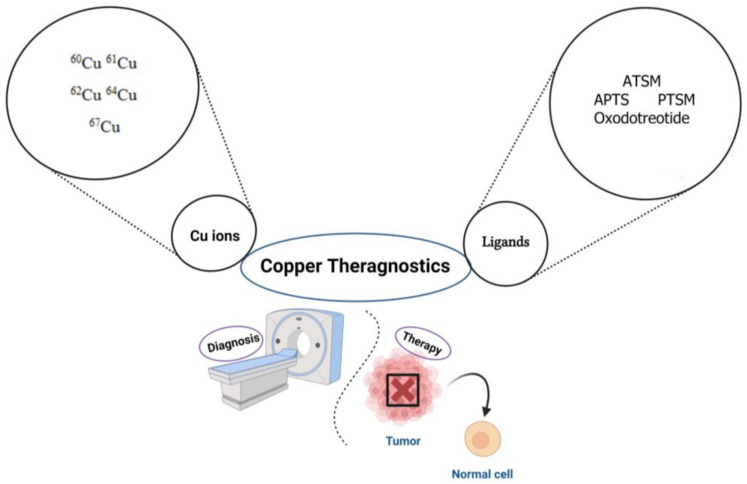
Major Cu ions and their ligands as promising theragnostic radionuclides for the diagnosis and treatment of different tumors. ^60^Cu, ^61^Cu, ^62^Cu are labeled with ATSM, APTS, PTSM. ^64^Cu is labeled with oxodotreotide (Dotatate). ^67^Cu as a β^+^ and γ-emitters can be used for both SPECT imaging and radiotherapy. ATSM: N4-methylthiosemicarbazone; APTS: 2-acetylpyridine thiosemicarbazone; PTSM: N4-methylthiosemicarbazone. Image created using Biorender (https://www.biorender.com/ accessed on 22 August 2023).

The combination of these emission routes makes this metallic radionuclide ideal for capturing high-resolution PET images, and in addition, this makes it an ideal therapeutic agent [89]. Μoreover, an interesting alternative to the single ^64^Cu radionuclide is the radionuclide pair ^64^Cu/^67^Cu. Copper-67 decays with beta emissions that are long enough to induce cell death in tumor cells. The coordination chemistry of this pair is also ideal for the linkage of a variety of chelators. Moreover, the ionic forms of copper, including the dicationic form Cu^2+^, are involved in several cellular processes, among which are cell proliferation and metastasis [90]. Therefore, the preparation of radiopharmaceuticals using copper has gained a lot of attention over the last decade (Figure 3).

### Biological Effects of Copper Ions in Cancer

Copper ions are involved in different biological processes during cancer progression. Normal and malignant cells exhibit significant differences in copper metabolism, and preclinical studies have demonstrated the multifaceted effects of copper on cancer development. The major mechanism of copper entry to both normal and malignant cells is through specific transporters, named human copper transporter 1 (hCTR1). Prior to entry, Cu^2+^ ions are reduced to Cu^+^ by reductases. Different studies have shown that hCTR1 transporters are overexpressed in tumors, including prostate cancer, breast cancer, melanoma, and glioblastoma, compared to normal cells [91,92,93]. Moreover, RNA-mediated knockdown of this transporter has resulted in reduced uptake of ^64^Cu in the tumor site, resulting in the inhibition of tumor development [94]. For this reason, this transporter has been proposed as an effective target for copper-based radiopharmaceuticals, with the aim of visualizing the tumor’s site.

## 4. Radiopharmaceutical Applications of ^64^Cu Isotope

The ^64^Cu isotope has been proposed as an effective metallic radionuclide for the development of theragnostic radiopharmaceuticals. ^64^CuCl_2_ is a promising agent for different types of tumors, including prostate cancer and glioblastoma. Ferrari et al. investigated the effect of this molecule against U87MG glioma cells using a xenografted GBM tumor mouse model. The investigators demonstrated that ^64^CuCl_2_ not only exhibits high affinity for GBM cells compared to normal cells, but it is also a potent anti-cancer agent, with the ability to inhibit cell proliferation after single- or multiple-dose treatments [95]. In another study performed by Qin et al., the use of ^64^CuCI_2_ as a theragnostic agent for malignant melanoma was investigated. The authors reported that ^64^CuCI_2_ showed high uptake in the studied melanoma cell lines, and the tumors were clearly visualized using ^64^CuCI_2_ PET imaging. It was also observed that this molecule could effectively reduce tumor growth in the same cell lines, thus acting as a promising theragnostic agent [96].

The use of ^64^CuCl_2_ as a PET imaging tool in a clinical environment was first introduced by Panichelli et al. back in 2016 in glioblastoma patients. The clinical study included 19 patients, of which 18 were diagnosed with glioblastoma and 1 with grade 2 astrocytoma. The findings of this study demonstrated that all 18 patients with high-grade glioma showed a significantly higher tumor uptake of ^64^CuCl_2_ compared to the patient with low-grade malignancy [97]. Moreover, recent studies have demonstrated that hCTR1-expressing tumor cells or xenografts show elevated ^64^CuCl_2_ uptake, meaning that this compound can be a helpful theragnostic tool for these types of tumors. For example, it has been found that ^64^CuCl_2_ can act as a promising imaging tool for the diagnosis of recurrent prostate cancer in small-scale human studies, with the additional benefit that no adverse effects were recorded in those participating in the studies. Back in 2018, Guerreiro et al. investigated the effects of ^64^CuCl_2_ on different prostate Ca cell lines compared to normal cells. Interestingly, their results showed that not only was the uptake of this compound higher in tumor cells, but it was significantly more cytotoxic against cancer cells, compared to the non-tumoral prostate cell line [98]. Recently, an inhibitor of SGK1, a serine/threonine protein kinase named SI113, has been investigated in combination with ^64^CuCl_2_ for its therapeutic role against glioblastoma cell lines. The results presented by the investigators show that co-treatment with SI113 and ^64^CuCl_2_ increases cell death and enhances the effects of ionizing radiation. Thus, such a combination could be the basis of developing novel theragnostic tools for the diagnosis and treatment of GBM [99].

As mentioned above, the radionuclide pair ^64^Cu/^67^Cu may be a promising theragnostic solution due to the concurrent PET imaging properties of ^64^Cu and the therapeutic potential of ^67^Cu. In a recent study, in which patients with unresectable multifocal meningioma were injected with [^64^Cu] Cu-SARTATE (with SARTATE being a somatostatin analogue chelated to the sarcophagine MeCOSar chelator) prior to treatment with [^67^Cu] Cu-SARTATE, nearly identical targeting of tumors was observed between patients, implying that this combination of copper radionuclides can be an effective theragnostic option [100]. In another study, in which the molecular target was the gastrin-releasing peptide receptor (GRPR), which is highly expressed in tumors like prostate cancer, a complex made of the pair of radionuclides ^64^Cu/^67^Cu, a bombesin (BBN) analogue, and a sarcophagine-based amine was used in a PC-3 xenograft prostate cancer mouse model. The results showed that [^64/67^Cu] Cu(SAR-BBN) displayed increased tumor uptake and retention, followed by a significant tumor growth inhibition [101].

## 5. Conclusions

In recent years, nuclear medicine has significantly contributed to the development of new tools for the concurrent diagnosis and treatment of different tumors. A wide variety of radionuclides can be linked to appropriate chelators and cytotoxic agents to design novel radiopharmaceuticals with theragnostic properties. The major advantages of this approach include improved diagnosis, reduced adverse effects to normal tissues, the elimination of multi-step procedures, and better patient care. Moreover, advancements in the field of nuclear medicine, such as the introduction of long-axial field-of-view PET/CT scanners, can improve current imaging and diagnostic tools by increasing sensitivity and resolution, as well as by reducing the level of injected radiopharmaceuticals while maintaining a high-resolution image quality. However, further research is needed to better understand the exact mechanism underlying the effects of theragnostic molecules in human cancer and to ultimately develop novel theragnostic agents with improved properties.

## Figures and Tables

**Table 1:** The physical characteristics and decay schemes of the theragnostic radioisotopes.

Radionuclides	Half Life (t_1/2_)	γ Ray (%)	Emission Type	E Average (keV)
^131^I	8.01 d	360 (6.2%) 720 (10.4%)	β^−^ (100%)	192
^18^F	1.83 h	511 (annihilation)	β^+^ (97%), EC (3%)	634
^68^Ga	68 min	511 (annihilation)	β^+^ (90%), EC (10%)	1899
^177^Lu	6.65 d	113 (6.2%) 208 (10.4%)	β^−^ 47.7 (11.6%) 111.7 (9.0%) 149.4 (79.4%)	134 (498 max)
^90^Y	2.67 d	Bremsstrahlung	β^−^	934 (2280 max)
^67^Cu	2.58 d	91 (7.0%) 93 (16.1%) 185 (48.7%)	β^−^	141
^64^Cu	12.7 h		β^+^ (17.5%), β^−^ (38.5%) EC (44.0%)	288 190 1675, 1346
^225^Ac	10.0 d	99.8 (1.0%)	α	α: (89.8%) 5732 5791 5793 5830
^223^Ra	11.44 d	154 (6%) 269 (14%)	α & β^−^ (3.6%)	α: (95.3%) 5606, 6819, 7386, 6623 β^−^: 445 (1370 max) 492 (1420 max)

**Table 2:** Molecular targets for theragnostics in GBM.

Molecular Target	Biological Process in GBM	Reference
Tenascin-C	Cell adhesion/extracellular matrix (ECM)	[61]
Epidermal growth factor receptor (EGFR)	Cell growth/survival	[62]
Chemokine receptor-4 (CXCR4)	Cell migration	[63]
Somatostatin receptor 2 (SSTR2)	Cell signalling/cell survival	[64]
Cadherin-3	Cell adhesion/extracellular matrix (ECM)	[65]
Neurokinin-1 receptor (NK1R)	Cell growth/survival	[66]
Integrin alpha-V beta-3 (αvβ3)	Angiogenesis	[67]
Fibroblast activation protein (FAP)	Inflammation	[68]
Poly (ADP-ribose) polymerase 1 (PARP1)	Cellular repair of DNA	[69]

GBM: Glioblastoma multiforme.

## Data Availability

Not applicable.
